# Stress Induced Activation of LTR Retrotransposons in the *Drosophila melanogaster* Genome

**DOI:** 10.3390/life13122272

**Published:** 2023-11-28

**Authors:** Polina A. Milyaeva, Inna V. Kukushkina, Alexander I. Kim, Lidia N. Nefedova

**Affiliations:** 1 Faculty of Biology, Lomonosov Moscow State University, 119234 Moscow, Russia; atemeda@mail.ru (P.A.M.); vladimirova-bph@yandex.ru (I.V.K.); aikim57@mail.ru (A.I.K.); 2 Faculty of Biology, Shenzhen MSU-BIT University, Longgang District, Shenzhen 518172, China

**Keywords:** LTR retrotransposons, abiotic stress, piRNA interference, regeneration

## Abstract

*Background:* Retrotransposons with long terminal repeats (LTR retrotransposons) are widespread in all groups of eukaryotes and are often both the cause of new mutations and the source of new sequences. Apart from their high activity in generative and differentiation-stage tissues, LTR retrotransposons also become more active in response to different stressors. The precise causes of LTR retrotransposons’ activation in response to stress, however, have not yet been thoroughly investigated. *Methods:* We used RT-PCR to investigate the transcriptional profile of LTR retrotransposons and piRNA clusters in response to oxidative and chronic heat stresses. We used Oxford Nanopore sequencing to investigate the genomic environment of new insertions of the retrotransposons. We used bioinformatics methods to find the stress-induced transcription factor binding sites in LTR retrotransposons. *Results:* We studied the transposition activity and transcription level of LTR retrotransposons in response to oxidative and chronic heat stress and assessed the contribution of various factors that can affect the increase in their expression under stress conditions: the state of the piRNA-interference system, the influence of the genomic environment on individual copies, and the presence of the stress-induced transcription factor binding sites in retrotransposon sequences. *Conclusions:* The main reason for the activation of LTR retrotransposons under stress conditions is the presence of transcription factor binding sites in their regulatory sequences, which are triggered in response to stress and are necessary for tissue regeneration processes. Stress-induced transposable element activation can function as a trigger mechanism, triggering multiple signal pathways and resulting in a polyvariant cell response.

## 1. Introduction

Transposition significantly affects the structure of the host genome and demonstrates a fundamental importance in the formation of genetic variability [1]. One of the most interesting types of transposable elements (TEs) for research is long-terminal repeat retrotransposons (LTR retrotransposons) since they have a common origin with retroviruses. Therefore, understanding the mechanisms of interaction between the host genome and LTR retrotransposons significantly contributes to understanding the mechanisms of interaction between the host genome and retroviruses. LTR retrotransposons insert into a new site through a replicative mechanism in which revertase synthesizes a new copy of TE on the matrix of its transcript and the integrase then inserts this copy into a new site [2].

The eukaryotic cell has a series of mechanisms to prevent transposition [3,4,5]. All of them are aimed at suppressing transcription and translation by RNA interference (mostly si- and piRNA). The highest activity of LTR retrotransposons in eukaryotes is observed in germline cells; therefore, the piRNA interference system, the main mechanism for suppressing TEs in germline cells, works intensively in these tissues [6]. *Drosophila melanogaster* is a model object on which the piRNA interference system has been investigated in detail. In *Drosophila*, this process is different in the somatic and generative ovary tissues.

In somatic tissues of *D. melanogaster*, piRNA precursors are transcripts of uni-strand clusters (aggregations of old copies of TEs transcribed in the antisense direction), such as *flamenco*. These long polyadenylated transcripts undergo alternative splicing and are sent to the protein complex on the outer mitochondrial membrane, Yb-body, where they are cut into smaller fragments and, after methylation, are transported to the nucleus in a complex with the PIWI protein, which finds the transcribed retrotransposon with the piRNA sequence and attracts proteins that provide a silencing of this region [6,7,8,9,10].

piRNA clusters, transcribed in two directions, undergo transcription initiation from non-canonical sites within the clusters by the RDC complex (Rhino, Deadlock, Cutoff) [11]. This complex not only drives RNA polymerase to start transcription from a non-canonic site but also prevents polyadenylation and capping of the transcript, due to which transcripts are delivered with no changes to the Nuage perinuclear area, where the Aub and AGO3 proteins start the ping-pong cycle. During this process, long transcripts of piRNA clusters interfere with TE transcripts, and their duplexes are cleaved into small fragments. This results in the formation of primary and secondary piRNAs, some of which serve as a template for TE RNA cutting and others for the cleavage of piRNA precursors [12,13,14]. In addition, several piRNAs from the ping-pong cycle are also used by PIWI to suppress TE activity at the transcription level [15,16,17]. Thus, the transposition activity of TEs and the subsequent mutation process directly depend on the stability of the piRNA system.

In addition to endogenous genomic factors, TE activity is often regulated by external stress conditions, such as heat and oxidative stress. It has been shown for different groups of organisms that certain TEs are able to increase their expression in response to biotic and abiotic stresses. For *Drosophila*, it has been shown that the *412* element copy number correlates with the temperature increase in wild populations of *Drosophila* [18]. It is also shown that in *D. melanogaster*, some TEs are able to increase expression, while others, on the contrary, decrease it under various types of stress. For example, four TEs increased their expression, while the other two decreased in response to dioxin, and only three of the studied TEs responded with an increase in expression under formaldehyde [19]. Thus, different TEs respond to different stresses individually. The hypothesis that TEs are adapted to stressful influences was proposed in the work of Barbara McClintock [20]. It is obvious that such adaptation occurs due to the accumulation of mutational changes, some of which may contribute to the conservation and spread of TEs. Another aspect of the problem is the possible role of TEs in the formation of the adaptive response of the organism to the influence of various stressors. While TE activation under strong stress conditions can lead to transposition, cause multiple damage to the genome, and lead to cell death, under weak stress conditions, TE activation can play the role of a trigger mechanism that starts several processes at once and provides a polyvariant reaction of a living cell in response to the stressor.

There are several molecular cascades involved in the response to various abiotic stresses, in particular, to oxidative stress: Jak/STAT, ERK, JNK, FOXO, MAPK, NF-kB, keap1/CncC, and PI(3)K/Akt, as well as proteins Hsp22, p53, and p38 [21,22,23]. Therefore, the presence of binding sites for certain transcription factors of various signaling cascades could explain the reason for individual TE responses to various oxidizing agents and other abiotic factors. Such binding sites were found with Chip-seq in many TEs of *Drosophila* and humans [24]. Under the influence of evolutionary processes, cis-regulatory elements of TE may become promoters, insulators, and enhancers for the genes of the host organism, in particular, *Drosophila*. And among these cis-regulatory elements, the vast majority belong to LTR retrotransposons [25]. Thus, the reasons for the activation of retrotransposons in response to stress may be associated with piRNA interference system functionality, the position of copies in the host genome, and the presence of their own binding sites for transcription factors.

In this work, we investigated the causes of changes in the expression of four well-studied LTR retrotransposons, *copia*, *gypsy*, *Tirant*, and *springer*, in response to oxidative and chronic heat stress. We analyzed the expression dynamics of selected TEs, piRNA clusters, and three markers of oxidative stress (*upd3*, *sid*, and *hsp22*). After that, we searched for conservative and unique insertions of these retrotransposons in the genomes of two strains, SS7K (the strain with impaired transposition control) and two wild-type strains, and analyzed the genomic environment of these insertions and their potential effect on the expression of these retrotransposons. We compared the change in the expression of two copies of *Tirant* and their genomic environment, searched for potential transcription factors binding sites, and then validated their conservatism among different copies.

## 2. Materials and Methods

### 2.1. Drosophila melanogaster Strains and Cultivation Conditions

The SS7K strain has a mutation in the *flamenco* locus that controls the transposition of the TE *gypsy* [26]; Canton-S is a wild-type strain; D32 is a wild-type laboratory strain from the collection of the Department of Genetics of Moscow State University. All strains were cultivated at 25 °C on the standard nutrient agar medium.

### 2.2. Induction of Oxidative and Chronic Heat Stress

To induce oxidative stress, adult seven-day-old females were incubated on a fresh nutrient agar medium containing 0.1 M ammonium persulfate (APS) for 24 h at 25 °C. After that, total RNA was isolated. To rest after stress, the flies were incubated on a standard medium for 24 and 48 h. To induce chronic temperature stress, adults were incubated on a fresh nutrient agar medium and left overnight at 25 °C. The next day, the parents were removed and the embryos were incubated at 29 °C until metamorphosis to imago. After hatching, the imago was incubated at 29 °C for 7 days, after which RNA was isolated from the females.

### 2.3. RNA Isolation, Reverse Transcription, and Real-Time PCR

RNA isolation was performed from the tissues of the ovaries, heads, and bodies using the ExtractRNA reagent (Evrogen, Moscow, Russia) in PBS buffer. RNA samples were treated with DNase I (Thermo Fisher Scientific, Waltham, MA, USA). The MMLV-RT Kit (Evrogen, Moscow, Russia) was used for reverse transcription. Reverse transcription was performed with a random primer for all samples (because the expression of most clusters in our experiment did not exceed the expression of low-copy TEs, and primers were designed for unique genome regions). PCR was performed in the presence of SYBR Green I (Evrogen, Moscow, Russia) on a MiniOpticon Real-Time PCR System (Bio-Rad, Hercules, CA, USA). In the experiment, we analyzed the relative expression of *gypsy*, *Tirant*, *copia*, and *springer* retrotransposons, *38C*, *20A*, *42AB*, and *flamenco* clusters, normalized to the expression of the *αTub84D*, *Rpl40*, and *EloB* genes (Table 1). Statistical processing of PCR results was performed using the Mann–Whitney test. Primers for the *42AB* cluster were designed earlier in [27]; primers for the *38C* and *20A* clusters were designed in [16]; primers for the *flamenco* cluster were designed for the unspliced form of the transcript according to the sequence from the NCBI database (Gene ID: 26067356). For primer design, we used the *flamenco* locus sequences that did not contain TEs. The forward primer was placed in the first exon and the reverse in the first intron. The selected primers cover the transcription region; however, they do not match the TEs in this cluster and therefore do not anneal to the cDNA of TEs.

### 2.4. DNA Isolation and Nanopore Sequencing

DNA isolation was carried out according to the standard method [28]. The DNA concentration in the sample was measured using a NanoDrop spectrophotometer (Peqlab, Erlangen, Germany). After that, the library was prepared according to the protocol of the Oxford Nanopore. The library was estimated using a NanoDrop spectrophotometer and loaded into the Nanopore MiniION Oxford sequencer. The sequencing data were mapped to the *D. melanogaster* reference genome (dm 6) in a virtual kernel (WSL) of Ubuntu for Windows 11 using miniconda2 (https://docs.conda.io/projects/miniconda/en (accessed on 3 December 2021)) with settings for reads obtained using Nanopore sequencing. The results were visualized using the IGV program [29]. For unique insertions of the studied TEs, we used the TLDR program [30]. The library of searched TEs was based on sequences from BDGP (https://www.fruitfly.org/p_disrupt/TE.html (accessed on 22 December 2021)). The results of the TLDR program were sorted by the length of the regions of new insertions matching the TE sequences, and long matches were checked using pairwise alignment in BlastN with the reference sequence [31]. The LASAGNA database [32], JASPAR, and TRANSFAC models of TF binding sites were used to search for binding sites with TF binding sites in the regulatory sequences of TEs. For analysis, only those binding sites were used that were predicted with a *p* < 0.001. The functions and expression patterns of TFs were checked in the FlyBase database [33]. The conservatism of the found binding sites was checked using multiple alignments in the Ugene [34]. We also searched for conservative TE insertions utilizing the Blast function in FlyBase and validated these positions in SS7K and Canton-S in IGV.

### 2.5. Evaluation of the Number of Copies of Transposable Elements by PCR

The reaction was run on a MiniOpticon Real-Time PCR System from Bio-Rad Laboratories. In the experiment, we analyzed the relative number of copies of the TE *copia*, gypsy, springer, and *Tirant* normalized on the number of single-copy genes *αTub84D* and *BoYb*. DNA from 20 females was used to determine the average copy number of LTR retrotransposons; 80, 40, and 20 ng DNA were taken into the PCR reaction, with two repeats for each concentration. After normalization on single-copy genes, the average number of copies of each TE between three dilutions for each strain was calculated.

## 3. Results

### 3.1. Transcription Analysis of LTR Retrotransposons under Oxidative and Chronic Heat Stress Conditions

**Figure 1:**
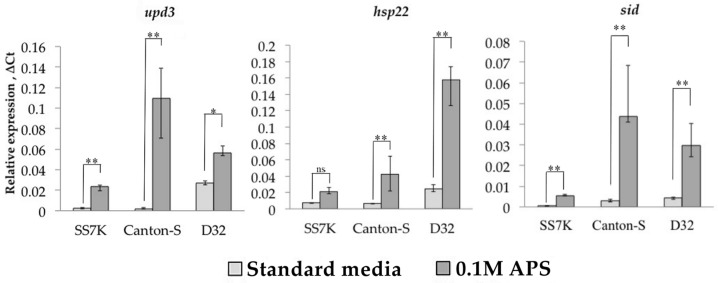
Relative expression of genes involved in the oxidative stress response during 24 h incubation on media with 0.1 M APS in females of SS7K, Canton-S, and D32 strains. (* *p* < 0.5, ** *p* < 0.01, according to the Mann–Whitney test; ns, not significant—statistically insignificant change).

We measured the expression of four LTR retrotransposons, *copia*, *gypsy*, *Tirant*, and *springer*, in response to oxidative and chronic heat stress. These TEs represent different groups of LTR retrotransposons with different copy numbers. Ammonium persulfate (APS) was used as an oxidizing agent, which activates oxidative stress-responding genes [35]. We measured the transcription level of three genes as a control for the induction of oxidative stress: *upd3*, *sid*, and *hsp22* (Figure 1). The expression of these genes increased significantly after a 24 h incubation of 7-day-old *Drosophila* females on 0.1 M APS in all three strains.

**Figure 2:**
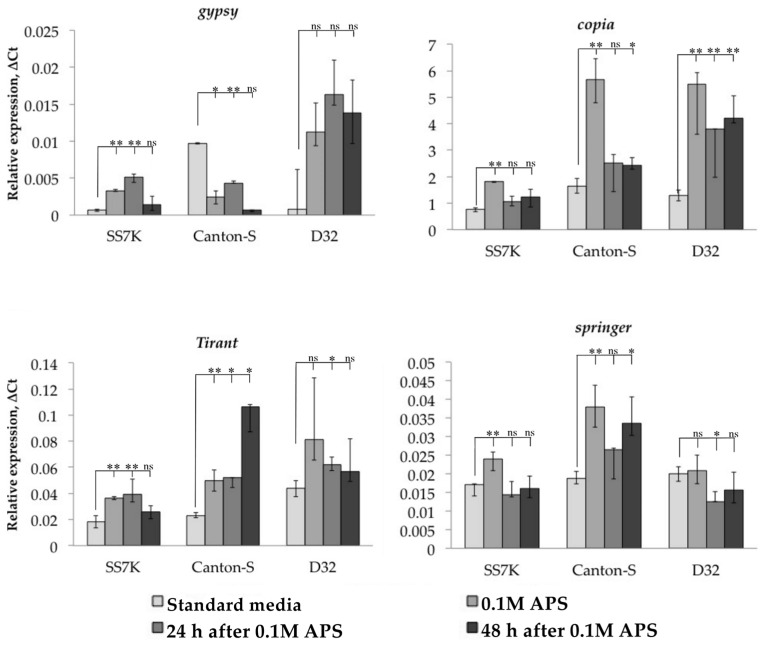
Dynamics of changes in TE expression in 7-day-old imago females exposed to APS and during rest after the stress (* *p* < 0.5, ** *p* < 0.01, according to the Mann–Whitney test; ns, not significant—statistically insignificant change).

After validating the induction of oxidative stress, we measured the transcription level of studied TEs in the same samples (Figure 2). TEs reacted to oxidative stress in most cases by increasing their expression; however, this was not a consistent pattern for all strains. Thus, most of the TEs in the D32 strain did not demonstrate a statistically significant change in expression in response to oxidative stress. At the same time, both without stress and during stress, we found a large scatter in the values of the expression level in different samples. This may indicate the genetic heterogeneity of this strain, which causes different levels of TE expression individually.

Additionally, we tracked the dynamics of the expression of particular retrotransposons 24 and 48 h after the stress was removed. We observed that LTR retrotransposon expression did not immediately return to the initial level in some cases. The differences between flies incubated on a standard media and flies exposed to an oxidizing agent were significant in the following 24 or 48 h of the rest after stress: *gypsy* and *Tirant* in SS7K did not decrease expression within 24 h; *Tirant* in Canton-S and *copia* in D32 did not decrease expression within 48 h.

**Figure 3:**
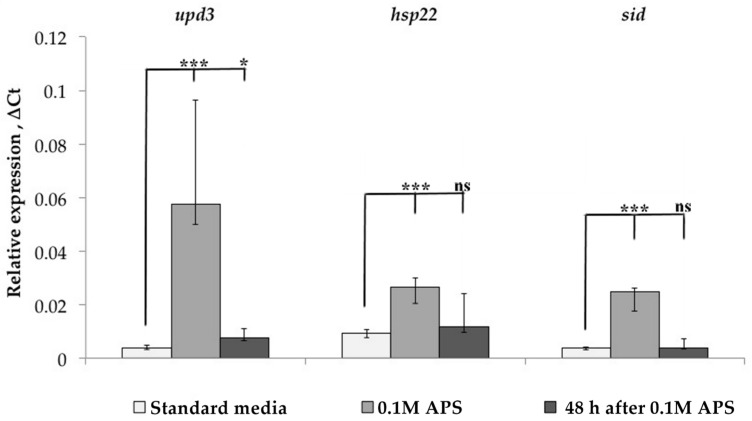
Dynamics of changes in the expression of genes-markers of oxidative stress in the Canton-S strain under treatment with APS and during rest (* *p* < 0.5, *** *p* < 0.001, according to the Mann–Whitney test; ns, not significant—statistically insignificant change).

Since TE transcription may depend on the piRNA interference system, the expression of various transcription factors, and the position in the genome of a particular strain, we analyzed each of these factors. First of all, we studied the dynamics of oxidative stress marker gene expression in the Canton-S strain before, under, and after stress exposure (Figure 3). The expression of these genes was significantly increased under oxidative stress but returned to a normal level 48 h after stress. At the same time, as was shown above, the expression of LTR retrotransposons at this time point in some cases was significantly higher than the baseline level. Thus, it can be argued that the regulation mechanisms of the studied genes and TEs are different since the expression of TEs after the onset of stress persists longer. Thus, TEs may have a mechanism that maintains their expression even during the recovery period.

**Figure 4:**
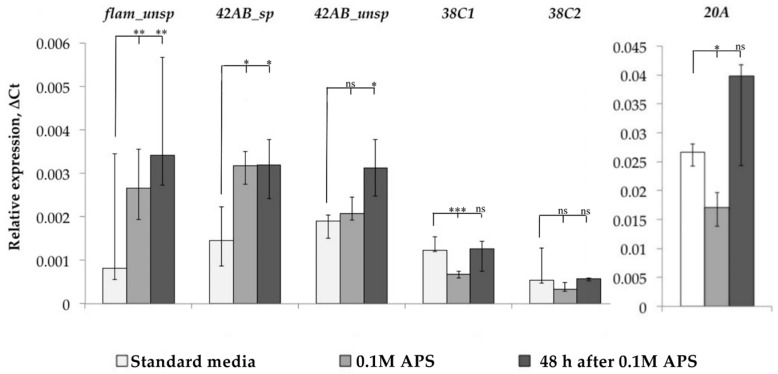
Dynamics of piRNA cluster expression. (* *p* < 0.5, ** *p* < 0.01, *** *p* < 0.001, according to the Mann–Whitney test; ns, not significant—statistically insignificant change).

After that, we evaluated the functionality of the piRNA interference system, the main mechanism of TE control in *Drosophila* ovaries, under oxidative stress. At least 20 proteins are involved in piRNA interference system functioning and their number is constantly being refined upwards [6]. Therefore, we decided not to study the expression of individual genes related to the piRNA interference system, but to evaluate its work by the level of piRNA cluster expression, i.e., the ratio of spliced and unspliced forms of transcripts of these clusters. We measured the dynamics of *flamenco*, *42AB*, *38C*, and *20A* cluster expression and evaluated the spliced and unspliced transcript form expression for cluster *42AB*, but such an analysis turned out to be impossible due to the absence of *flamenco* transcript spliced forms in the Canton-S strain, as well as in the D32 strain. For clusters *20A* and *38C*, we analyzed only unspliced transcript forms (Figure 4).

As a result, we found that *flamenco* and *42AB* cluster expression was increased in response to oxidative stress, and we detected an increase in the amount of spliced forms in the case of the *42AB* cluster, which can affect the functionality of the piRNA interference system since the mechanisms that prevent the splicing of this cluster transcripts are active under normal conditions because their introns contain sequences that are the source of piRNA. Analyzing the other two clusters, we did not detect an increase in *20A* cluster expression, while *38C*, on the contrary, reacted with a decrease in expression. Thus, the overall change in piRNA interference clusters in some cases (*flamenco* and *42AB*) remained unchanged even 48 h after stress, and TE expression in response to stress may be associated with a misregulation of individual piRNA interference system components.

**Figure 5:**
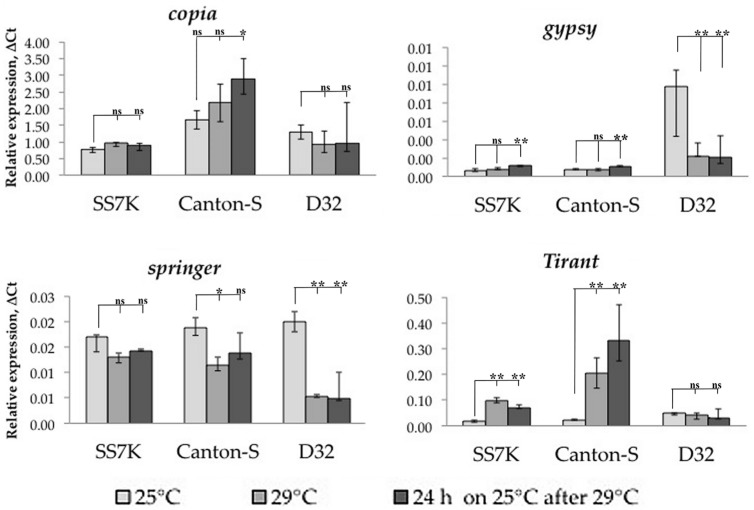
Dynamics of LTR retrotransposon expression in seven-day-old adult females under chronic heat stress and rest (* *p* < 0.5, ** *p* < 0.01, according to the Mann–Whitney test; ns, not significant—statistically insignificant change).

To induce chronic heat stress, we incubated flies at 29 °C. Since this stress is poorly studied, and marker genes for chronic heat stress are not known (in this situation, we do not consider heat shock), we estimated the heat stress presence by male sterility. Measuring LTR retrotransposon expression under chronic heat stress, we found that only *Tirant* significantly increased expression in the SS7K and Canton-S strains, while the expression of other TEs does not change under these conditions, and the expression of *Tirant* is maintained at a high level even after stress (Figure 5).

Therefore, changes in TE expression under stress conditions are not always regular. Some TEs respond to only one stressor, while others are capable of responding to a range of extreme conditions with different effects.

### 3.2. Analysis of the Relationship between Transposable Element Copy Number and Their Position in the Genome with Their Transcription Activation

Obviously, it is unreasonable to compare the transcription of different TEs between strains because there are many factors that reduce the effectiveness of this approach. These factors will be discussed below. However, we can assume that the expression level of each individual TE may be related to the copy number in the genome: the more copies the higher the expression level. The expediency of TE expression normalization on its copy number is questioned. Since it is impossible to choose a universal primer that would anneal on all studied TE copies, we can only estimate the total expression of TEs that this primer is complementary to. In the same way, by isolating DNA from 20 flies of each strain, we estimated the number of copies whose expression was measured with RT-PCR (Table 2).

Comparing this data with the TEs’ expression histograms, we observe that their expression may depend on the number of copies, but this correlation is not linear. Therefore, normalization of TE expression on the number of its copies in the genome is not an effective approach.

Expression of TEs under normal and stress conditions can also be determined by the position of their copies in the genome of studied strains. Therefore, we analyzed the position of TE copies in the studied genomes, where they demonstrated a significant expression change in response to environmental conditions: Canton-S and SS7K. For position analysis, we first chose the TE copies that were absent in the reference genome, which are, therefore, new and, probably, functionally active. The next requirement to select copies was that they contain the sequences of the used primers. All insertions were in intergenic space, introns, or 3′-UTR of genes (Table 3).

Most of the unique TE insertions localized in genes were not involved in the stress response. Not one of the insertions in the genes induced under stress was collinear to the gene transcription direction. Therefore, in this case, the effect of the position should be minimal.

We also identified the position of the TE copies that were present in the reference genome and analyzed the presence of these copies in the Canton-S and SS7K genomes (Table 4). However, most of these regions in the sequenced genomes did not contain uniquely mapped reads or had poor coverage. But we also frequently observed the absence of TE sequences in reads, probably due to the fact that these insertions are unique in the reference genome and may not be present in our strains. Almost all of the TE copies presented in the reference genome were heterozygous in our strains.

After analyzing the insertions of studied LTR retrotransposons common to the reference genome and these two strains, we revealed the same trend as for unique insertions. TE copies were located in the intergenic space or in the introns of the genes, as a rule, and most of them were located in a chain complementary to the direction of gene transcription. Most of the genes that contained copies of the TEs, according to FlyBase, did not increase their expression in response to oxidative stress or heat shock; almost all of the insertions were heterozygous. We did not find any copy of the *springer* of *gypsy* in stress-activated genes. Therefore, in this case, we can assume that LTR retrotransposon expression is not determined by its insertion site. Moreover, normalizing expression to the number of copies is impractical, because each RNA sample contains the number of transcripts that correspond to a different number of original copies of TEs due to strain heterogeneity.

**Figure 6:**
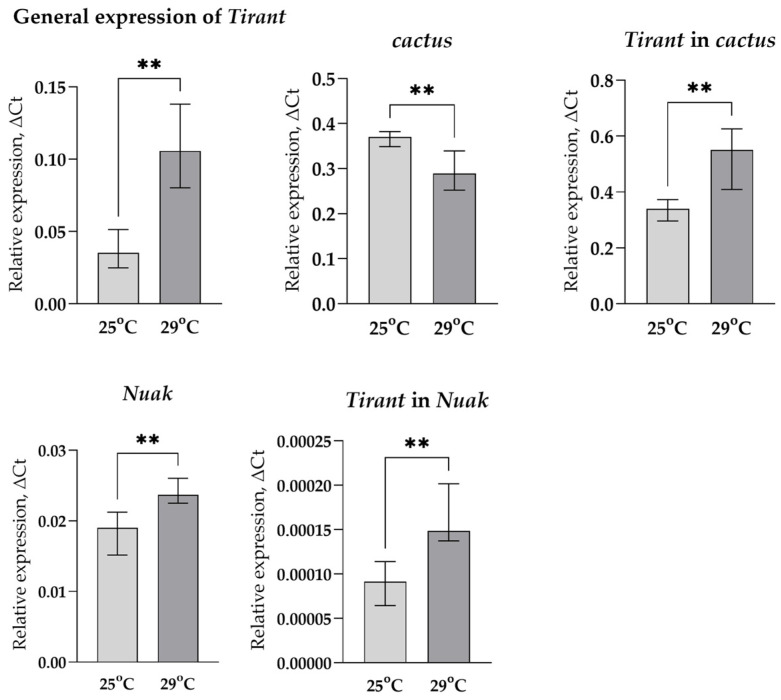
Changes in the expression of LTR retrotransposon *Tirant*, *cactus*, and *Nuak* genes in response to chronic heat stress (** *p* < 0.01, according to the Mann–Whitney test).

To get an idea of how the position of the TE might affect its expression, we took two different variants of the *Tirant* (according to the sequence of our laboratory strain SS7K), designed allele-specific primers for these copies, and compared the change in their expression in response to stress impact with changes in the expression of the genes in which these TE variants were inserted: *Nuak* and *cactus*. Since the change in the total expression of *Tirant* is higher under chronic heat stress, the chance to catch the change in the expression of individual copies under these conditions is much more probable than under oxidative stress, so we analyzed its expression only in the case of chronic heat stress (Figure 6).

We observed an increase in *Nuak* expression and a decrease in *cactus* expression under chronic heat stress, while the total expression of *Tirant* and both of its copies increased. This suggests that the *Tirant* probably has its own binding sites of transcription factors (TFs), which activate its transcription in response to stress.

### 3.3. Search for Transcription Factor Binding Sites in the Regulatory Regions of LTR Retrotransposons

To verify the presence of TF binding sites in the studied retrotransposons, we took their LTR and 5′-UTR sequences and analyzed them for the presence of potential binding sites using the LASAGNA resource. As a result, we obtained a list of potential TF binding sites, among which we were interested in stress-induced TFs (the same transcription factors were responsible for early embryonic development of *Drosophila*). After that, we performed multiple alignments of recent TE insertion sequences using Ugene and checked the conservatism of these binding sites (Table 5).

Thus, all four TEs demonstrated conserved binding sites of TFs activated in response to oxidative and chronic heat stress. Although, for low-copy LTR retrotransposons *gypsy* and *Tirant*, this analysis is not clear. The presence of such sites, as well as the activation of individual alleles of *Tirant* in response to chronic heat stress, indicates that the regulation of retrotransposons under extreme conditions depends on the features of the regulatory sequences of their individual copies and does not always depend on the position in the genome. Moreover, impaired splicing of transcription of double-stranded clusters, such as *42AB*, also may be a consequence of stress and be one of the reasons for LTR retrotransposon repression weakening.

## 4. Discussion

We observed the activation of individual LTR retrotransposons under oxidative stress caused by ammonium persulfate, as well as under chronic heat stress. Moreover, different TEs were activated to different degrees; however, the activation itself mostly did not depend on the initial state of the piRNA interference system. For example, in the wild-type strains, D32 and Canton-S, the expression of *copia* increased no less than in the strain with a mutation in the *flamenco* cluster, SS7K. We also registered an increase in *Tirant* expression regardless of the initial state of the piRNA interference system under chronic heat stress.

We could assume that TE transcription activation depended on changes that occur in the piRNA interference system during stress exposure. However, we have shown that individual clusters (uni-strand *flamenco* and dual-strand *42AB*) also increased their expression, which might be a response to elevated TE activity under stress conditions. According to the modern concept, piRNAs can be cleaved from transcripts of repressed TEs; therefore, we considered clusters as piRNA sources in the genome (since it is impossible to validate the transcription of piRNA sources that are represented as satellite copies of retrotransposons) [36]. Also, using the *42AB* cluster, we have shown that stress leads to a misbalance in the splicing mechanism and disruption of piRNA biogenesis. We cannot definitively conclude whether the imbalance between spliced and unspliced forms is associated with the disruption of the functioning of the RDC complex genes under conditions of oxidative stress. There are other factors that influence piRNA splicing. The heat stress protein, Hsp70, has previously been shown to play a role in piRNA biogenesis; during heat shock, this protein localizes to Nuage, where it binds to AGO3 and Aub. The release of this protein from the complex occurs in three days after stress is removed [37]. However, we observed that not all TEs that were activated in response to oxidative stress demonstrated the same activity under chronic heat stress. It is known that mutations of the main genes involved in piRNA interference (*aub* and *ago3*) do not lead to an increase of absolutely all TEs’ expression but only to several retrotransposons (*gypsy*, *roo*, *copia*, *Tirant*, and *blood*) [38]. Thus, the regulation of TEs controlled by the same piRNA interference system differs under a range of conditions. This may indicate the existence of alternative regulatory subsystems for different TEs or the repression of individual components of the piRNA interference system under stress conditions. Other researchers have shown a similar behavior of TEs under different types of stress: different chemical and physical agents cause an increase in different TE expressions, and the number of activated retrotransposons rises with stress intensification [19].

The second problem investigated in this work was the applicability of the normalization of TE expression on TE copy number. We have seen that the expression of LTR retrotransposons did not linearly correlate with the amount of TE copies. Moreover, laboratory strains that are not subjected to selection or gene drift for a long time become a polymorphic cause of the insertion of new TE copies; therefore, the amount of RNA was transcribed from an initially different number of TE copies in each sample, which also prevents the normalization of expression to the average number copies in the genome. Also, using the expression level of individual insertions of *Tirant*, we observed that the change in the total expression of this TE was more significant than the change in the expression of individual copies. Thus, taking into account the potential individual features of individual copies regulation, we can conclude that the normalization of any TE expression on the number of its insertions can result in misrepresentation.

We considered two hypotheses for the possible regulation of TE expression: the influence of the genomic environment and the presence of transcription factor binding sites in TE regulatory regions. After analyzing the positions of TE insertions in the SS7K and Canton-S genomes, we did not find insertions that could be affected by the genomic environment: almost all insertions were in intergenic space, and most genes that contained insertions in introns, according to the FlyBase, were not responsible for stress response. Next, we selected allele-specific primers for two copies of *Tirant* in the SS7K strain, since we implemented sequencing of this stain in our laboratory, and, therefore, we could detect these copies using PCR. After the induction of chronic heat stress (*Tirant* increased its expression higher in these stress conditions), we observed that the genes in which the insertions were located did not respond to stress. But the general expression of *Tirant*, as well as the expression of its individual copies, changed significantly. Thus, most TE insertions are capable of self-regulation rather than being co-expressed with the genes in which they are located.

To test the conservatism of TFs’ binding sites in LTRs and 5′UTRs, we performed a multiple alignment of all new insertions revealed in analyzed genomes and found that some sites predicted by the LASAGNA program were indeed conserved among the new TE insertions. Despite the fact that all these TFs are activated in response to stress, they have another role in explaining the maintenance of LTR retrotransposon expression. The role of these TFs is in the development of the organism, regulation of differentiation processes, and maintenance of stem cells. For example, HSF is necessary for early larval development and oogenesis, as well as the development of *Drosophila* [39]; *cad* is important for the determination of the anterior–posterior axis of the embryo, as well as maintaining stem cells in the gut [40]; *slbo* and *Trlare* are involved in cell migration during oogenesis [41]; and *Lag-1* regulates differentiation of stem cells [42]. Thus, TE activation under stress does not occur directly in response to stress but under the influence of tissue regeneration processes that are triggered by this stress. This conclusion is also based on the fact that Hsp70, which, like TE, was expressed in the cell for three days after stress was removed, is triggered by HSF, which is the activator of heat shock proteins [37,43]. *Cad* demonstrates a pleiotropic effect and is important for stress response in adults: it prevents the production of antimicrobial peptides in the hindgut, which are induced under stress conditions by *Relish*. *Relish* is one of the participants in the IMD pathway and is bound by negative feedback with *cad* [44]. The activation of antimicrobial peptides themselves is associated with the IMD and Jak-STAT pathways [45]. Both pathways respond to stress under ammonium persulfate treatment [35].

The presence of TF binding sites explains why LTR retrotransposons are active in generative tissues during the development of the organism, respond to stress, and do not decrease their expression immediately after the removal of the stress inducer. The reason for the non-total increase in LTR retrotransposon expression in response to various poisons and oxidizing agents precisely underlies the specific action of different oxidants, which leads to the involvement of different molecular cascades in response and, consequently, several LTR retrotransposons.

We assume that the primary reason for the activation of TEs in response to stress is the presence of TF-binding sites in their regulatory regions. Due to the presence of these sites, the cell moderates TE activation during the regeneration of damaged tissues. But to prove it, it is necessary to conduct ChIP-seq experiments. The position of individual copies in the genome is a minor reason for TE activation. However, the TE activation process should also be beneficial for the host organism. This suggests that only those TEs that contain binding sites for specific TFs in their regulatory regions were selected during evolution. Apparently, the co-activation of TEs with stress response genes contributes to the overall mobilization of the stress response, which was previously shown by other researchers [24,25,46]. However, in this case, we observed that TE expression continues for the next 48 h following stress removal. In addition, all TFs, for which the binding sites are present in the studied TEs, respond not only to stress but also take an active part in early embryonic development; thus, it is possible that the process leading to an increase in TE expression is not so much the stress itself but the recovery process.

The TEs underwent extensive selection, during which their relationship with the host genome evolved from parasitic to mutually beneficial. Therefore, the *L1* element in mammals is necessary for embryonic development and neurogenesis, as it regulates the expression of many genes [47]; other TEs participate in tissue-specific regulation of transcription [48]. In addition, TEs can play the role of ncRNA sources, which suppress the activity of certain genes, such as miR-431 [49,50]. This ncRNA is required for axon regeneration after injury. The effect of active axon growth after damage was also observed in the nematode with a mutation of the *piwi* gene, a core participant of the piRNA interference system, which suggests that this process is conservative [51]. Also, mosaicism caused by TE transpositions during the development of the nervous tissue is also a necessary condition for the normal functioning of the nervous system. Therefore, TEs studied in our case and in a number of other similar studies are TEs that have already passed through several stages of domestication and are controlled by the host genome.

## 5. Conclusions

Under stress, TE activation can lead to transposition, causing extensive genomic damage and resulting in cell death. On the other hand, TE activation can serve as a trigger mechanism, driving multiple processes simultaneously and providing a multivariate response of a living cell to negative factors. Thus, the activation of TEs during stress is not associated with the resistance of the cell to an oxidizing agent or any other stressor, but with regeneration processes that start at this moment and continue after stress. The regulation of TEs in response to stress and during development seems to be the same. This is a matter for further research.

## Figures and Tables

**Table 1:** Primers used to evaluate TEs, genes, and piRNA cluster expression, as well as to count the copy number of the studied transposable elements.

Name	Forward Primer	Reverse Primer
Genes		
*αTub84D*	5′-GTGCATGTTGTCCAACACCAC-3′	5′-AGAACTCTCCCTCCTCCATA-3′
*EloB*	5′-GCACAAACATACACACTCACG-3′	5′-TTTCCTACTTCGCTTGCACC-3′
*RpL40*	5′-CTGCGTGGTGGTATCATTG-3′	5′-CAGGTTGTTGGTGTGTCC-3′
*hsp22*	5′-CTTTTCACGCCTTCTTCCAC-3′	5′-GTGAGTTTGTAGCCATCCTTG-3′
*sid*	5′-GGAAGTGTTCAAGCGATTG-3′	5′-AGCAGATACAACGTCTGGTG-3′
*upd3*	5′-AACGGCCAGAACCAGGAATC-3′	5′-GAGAGGGCAAACTGGGACAT-3′
*cactus*	5′-GATCTCAGCGAGGAGATAGTC-3′	5′-CTCCCTCCTCTTTCTCCTGC-3′
*Nuak*	5′-GGTTCCTGTTTCCCAGTTACTC-3′	5′-ATCACTTTGGTGGCATCCTTTT-3′
*BoYb*	5′-CCCAAGTTTCTCATGGTTTC-3′	5′-ACGAACTGCTCCCGAATATG-3′
Clusters		
*flanemco-unspliced*	5′-CATCAGCTCAGCAGCAGTGTA-3′	5′-GACTTAACACTTACCGCTTGAAA-3′
*42AB-spliced*	5′-GCAGTTGCCGTCTCTCCT-3′	5′-TGGGTCAAAGTGCAGCAGTT-3′
*42AB unspliced*		5′-TACGGGAATATAATCGCAGCAGTT-3′
*38C1*	5′-AATGGCTAGTTCGCTACCAGACAG-3′	5′-CGGGTCTTCTCTCAAACGCAATC-3′
*38C2*	5′-CACAAAATGGCCCGCTGGAAA-3′	5′-AGCCAAACCCTGTGTTGTGA-3′
*20A*	5′-GCCTACGCAGAGGCCTAAGT-3′	5′-CAGATGTGGTCCAGTTGTGC-3′
Retrotransposons		
*gypsy*	5′-CTGCTGAAAGACGGCATTATC-3′	5′-AGAACTTTGCCTTGCCCAGAT-3′
*Tirant*	5′-AACGCTATTCATTCTGCAAC-3′	5′-AGGGTTCTCCTAACTACGTC-3′
*copia*	5′-CTTCAGTGATGGACAACTG-3′	5′-CAGTGTAATCTCATGGTCAT-3′
*springer*	5′-CTAAATTCGCCATGGTACAGC-3′	5′-AGCCGAGGAGTAAATGAGTA-3′
*Tirant* in *cactus*	5′-GGGACCTTTCGCCTCAAC-3′	5′-GCCACTATTGTCTGCGATTT-3′
*Tirant* in *Nuak*	5′-GCCTGAGTTAACAAAGGTGAAC-3′	5′-GTAGGAATCAAAAAACTACACAACC-3′

**Table 2:** Number of TE copies in the SS7K, Canton-S, and D32 strains.

Strain		gypsy	copia	Tirant	springer
	Transposable Element				
SS7K		3	44	5	6
CantonS		3	55	4	6
D32		2	46	11	6

**Table 3:** Unique insertions of LTR retrotransposons *gypsy*, *copia*, *Tirant*, and *springer* in the SS7K and Canton-S.

LTR Retrotransposon	Gene, Contaning TE Insertion	Insertion Position in the Gene	Collinearity with the Direction of the Gene Transcription	Response of Genes to Oxidative Stress and Heat Shock (According FlyBase)
SS7K				
*copia*	*lr42a*	Intron	No	No
	*IRSp53*	Intron	No	No
	*bnl*	Intron	No	No
	*CG34353*	Intron	Yes	No
	*Pde9*	Intron	No	Moderate to both stresses
*Tirant*	*cactus*	3′-UTR	No	High to both stresses
	*Nuak*	Intron	No	No
Canton-S				
*copia*	*CG15431*	Intron	Yes	No
	*CG17684*	Intron	Yes	No
	*SLO2*	Intron	No	No
	*Pdk1*	Intron	No	Moderately high to stresses
	*dpr6*	Intron	Yes	No
	*CG45782*	Intron	No	No
*gypsy*	*CG42346*	Intron	No	No
*springer*	*eyes*	Intron	Yes	No
	*dnc*	Intron	No	No
	*CG30389*	Intron	No	High to oxidative stress, moderately high to heat shock
	*CG33970*	Intron	No	No

**Table 4:** Insertions of the LTR retrotransposons *gypsy*, *copia*, *Tirant*, and *springer* common to reference genome and SS7K and Canton-S strains.

LTR Retrotransposon Insertion		Expression in Stress Conditions According to FlyBase		Strains	
Position in the Gene	Collinearity with the Direction of the Gene Transcription	Oxidative Stress	Heat Shock	SS7K	Canton-S
*copia*					
*CG3726* (intron)	No	Moderate		+	+
*toc* (intron)	No	Moderately high	Moderate	+	+
*for* (intron)	No	Moderately high	Moderately high	+	+
*Ir40a* (intron)	Yes			+	+
*CadN2* (intron)	Yes			+	+
*CG44623* (3′UTR)	No				
*mbl* (intron)	No				
*Nipped-A* (intron)	No				
*sallimus* (intron)	Yes				
*bbg* (intron)	No				
*Snap25* (intron)	No	Moderate		+	+
*Lasp*, *CG43954* (intron)	No	High	High	+	+
*Eip63E* (intron)	Yes	Moderate		+	+
*Myo81F* (intron)	No				
*beat-Vb* (intron)	No				
*CG34347* (intron)	Yes				
*Gprk2* (intron)	No	Moderate	Moderate	+	+
*Tirant*					
*CG42534*(intron)	No				
*CG42238*(intron)	No				
* Cipc * (intron)	Yes				
* SNF4Aγ * (intron)	No	Moderately high	Moderate	+	+
* Raf * (intron)	No	Moderate			+
* CG34417 * (intron)	No	Moderately high	Moderate		
* CG32486 * (intron)	No	Moderately high	Moderately high		+
* skd * (intron)	No	Moderate	Moderate	+	+
*Shab* (intron)	No	Moderate			
* psq * (intron)	No	Moderately high	Moderately high		+
* unc-5 * (intron)	Yes				
* chn * (intron)	Yes	Moderate	Moderately high		
* CG11360 * (intron)	No				
* raw * (intron)	Yes				
* Fs(2)Ket * (intron)	No				

**Table 5:** Transcription factor binding sites in new LTR retrotransposon copies.

LTR Retrotransposons	Transcription Factors	Binding Site
LTR		
*copia*	HSF(M00164)	AGAGTGGTATTCTCT
*gypsy*	HSF(M00166)	CCTCGAGCAATGCAT
	Bcd(T00063)	ACCTAATCTCCA
	Lag1(MA0193.1)	CTACTGG
*Tirant*	Cad(T00079)	AGCATAATGA
	Cad(T00079)	GGCCGAATG
	Deaf1(MA0185.1)	AGCATTCGGCCGGAA
	h(MA0449.1)	GCCACATGCC
	slbo(MA0244.1)	ATTACACA
*springer*	Cad(T00079)	GGCCAAATGC
5′UTR		
*copia*	Zeste(M00283)	GAATTTGAGTGAAAA
*Tirant*	Cad(T00079)	GTCATAATTT
	dl(M00043)	CGGTATGTCCA
	CF2-II(M00012)	CTATATACA
	CF2-II(M00012)	ATATATGTG
	slbo(MA0244.1)	ATTGCACA
	slbo(MA0244.1)	ATTGCAGA
	Cad(T00079)	GTACTAAAGA
	tll(MA0459.1)	TAAAGCCAAA
	tll(MA0459.1)	AGAAGTCGAC
	CF2-II(M00012)	CTATATGCA
	Dll(MA0187.1)	TAATTAC
*springer*	cad(MA0216.1)	CTTATTG
	ovo(MA0126.1)	TGTTACTGT
	dl(M00120)	TAAGAAAATCG
	dl(M00043)	CGGCATTTCCA

## Data Availability

Additional information regarding the manuscript will be welcomed by the authors.
